# Gene therapy benefits in a small cohort of symptomatic 5q spinal muscular atrophy patients and real world pediatric outcomes

**DOI:** 10.1016/j.jped.2026.101597

**Published:** 2026-09-05

**Authors:** Andresa R.B.V. Santos, Lisiane S. Ferreira, Tamires S. Santos, Juliana F. Mazzeu, Ney C.A. Boa Sorte

**Affiliations:** a Universidade de Brasília (UnB), Hospital Universitário de Brasília (HUB), Brasília, DF, Brazil; b Universidade de Brasília (UnB), Brasília, DF, Brazil; c Hospital Universitário Professor Edgard Santos, Universidade Federal da Bahia (UFBA), Salvador, BA, Brazil

**Keywords:** Spinal muscular atrophies, Gene therapy, Child, Motor activity, Respiratory insufficiency

## Abstract

**Objective:**

To characterize clinical outcomes of gene replacement therapy administered after symptom onset in a small group of children with 5q spinal muscular atrophy (SMA) and to describe its impact on motor function and supportive care needs in real-world practice.

**Methods:**

Observational cohort study with retrospective and prospective data collection at a single pediatric center. Children with molecularly confirmed type 1 or type 2 5q SMA who received onasemnogene abeparvovec and remained in multidisciplinary follow-up were consecutively included. Due to the small sample (n = 7) and patient heterogeneity, analysis was exclusively descriptive.

**Results:**

Seven symptomatic children were evaluated (six type 1, one type 2). After treatment, motor milestones showed localized improvements; all type 1 patients achieved head control and sitting with support, and 83.3% achieved independent sitting. The type 2 patient achieved independent ambulation. Adverse events occurred in 85.7% of patients and were mostly mild to moderate, though one case of thrombotic microangiopathy required dialysis with favorable recovery. Despite motor gains, prior and simultaneous use of other therapies (nusinersen, risdiplam) acted as confounding factors, and most children remained dependent on noninvasive ventilation and gastrostomy.

**Conclusions:**

In this small and heterogeneous cohort, gene therapy after symptom onset was primarily associated with clinical stabilization and limited motor milestone acquisition, while established respiratory and nutritional impairments persisted. These results emphasize the need for realistic treatment counseling and reinforce the importance of early diagnosis.

## Introduction

5q spinal muscular atrophy (SMA) is a genetic neuromuscular disorder with autosomal recessive inheritance, characterized by progressive degeneration of motor neurons in the spinal cord and brainstem, leading to muscle weakness, respiratory impairment, and bulbar dysfunction [1]. It is one of the leading genetic causes of infant mortality associated with neuromuscular disease, with an estimated incidence ranging from 1:6000 to 1:11,000 live births [2,3].

The pathophysiology of 5q SMA is related to homozygous deletion of the *Survival Motor Neuron 1* (*SMN1*) gene, responsible for the production of the survival motor neuron (SMN) protein [4]. The *Survival Motor Neuron 2* (*SMN2*) gene, a paralog of *SMN1*, partially compensates for SMN protein production. The number of *SMN2* copies is one of the main modifiers of clinical severity, although it does not independently determine disease phenotype [5].

In recent years, the management of 5q SMA has been profoundly modified by the introduction of disease-modifying therapies, including nusinersen, risdiplam, and gene therapy with onasemnogene abeparvovec-xioi [6., 7., 8.]. The latter consists of a single infusion of an adeno-associated viral vector carrying a functional copy of the *SMN1* gene, with the potential to alter the natural history of the disease, particularly when administered early [8].

However, much of the available clinical trial evidence on gene therapy involves restrictive inclusion criteria, which limits the extrapolation of results to real-world clinical practice, especially in middle-income countries where treatment is frequently initiated well after symptom onset [9,10]. Systematic observational data from small, diverse cohorts remain scarce but essential to understanding the partial clinical stabilization and the ongoing healthcare needs of these patients. In this context, the aim of this study was to describe the motor, respiratory, nutritional, and safety outcomes in a small group of symptomatic children with 5q SMA treated with onasemnogene abeparvovec-xioi in a real-world setting.

## Methods

This was an observational cohort study with retrospective and prospective data collection, conducted at a tertiary pediatric referral center, with follow-up through periodic clinical evaluations between March 2024 and June 2025.

This descriptive study clinical cohort comprises a select sample of seven patients from the Federal District (DF) and surrounding areas, without a concurrent control group. The cohort intentionally includes different phenotypes of the disease (six patients with type 1 SMA and one with type 2 SMA) and encompasses patients who had been submitted to prior or simultaneous disease-modifying therapies (nusinersen and risdiplam). Due to the small sample size and patient heterogeneity, the statistical approach is restricted to an exclusively descriptive analysis.

Given the exploratory and descriptive nature of this study, it features a very small sample size (n = 7) and notable patient heterogeneity, including both type 1 and type 2 phenotypes. Crucially, the prior and simultaneous use of other disease-modifying therapies (nusinersen and risdiplam) represents a significant confounding factor. Furthermore, the lack of a concurrent control group restricts the isolation of the effect of the gene therapy itself, and thus results are limited to descriptive analysis.

Eligible participants were children with a molecularly confirmed diagnosis of type 1 or type 2 5q SMA who received a single infusion of onasemnogene abeparvovec-xioi and remained under multidisciplinary follow-up with the study team. All families were initially contacted by the responsible clinical team, and inclusion occurred after written informed consent from legal guardians. Patients were excluded if guardians declined continued follow-up, if follow-up was lost before the first clinical assessment, or if death occurred prior to the initial data collection. All individuals meeting eligibility criteria during the study period were consecutively included.

Data were obtained from medical records, clinical care documentation, and prospective clinical assessments conducted by the local team. Collected variables included demographic and perinatal characteristics, genetic information, therapeutic history, adverse events, need for respiratory and nutritional support, and clinical and functional outcomes. Data management was performed using a secure electronic data capture platform (Research Electronic Data Capture – REDCap), ensuring traceability, controlled access, and data security.

Motor function was assessed using standardized scales according to age and functional level: the Children’s Hospital of Philadelphia Infant Test of Neuromuscular Disorders (CHOP-INTEND), the motor milestones section of the Hammersmith Infant Neurological Examination (HINE-2), and the Hammersmith Functional Motor Scale Expanded (HFMSE). Assessments were performed by trained and certified evaluators. For analysis, the first motor assessment performed by the participating team after gene therapy infusion was considered the baseline post-treatment evaluation.

The primary outcome was motor functional evolution after gene therapy infusion, assessed by motor milestone acquisition and scores on standardized motor scales. Secondary outcomes included adverse events, need for respiratory support, and nutritional status.

Standardized clinical evaluations were performed, including a complete neurological examination with assessment of muscle tone and strength, deep tendon reflexes, motor milestones, and investigation of orthopedic deformities. Respiratory function was characterized by the need for and type of ventilatory support, use of airway clearance devices, and presence of tracheostomy. Swallowing and nutritional status were evaluated according to feeding route, use of gastrostomy or enteral tube feeding, and oral intake capacity.

Adverse events were defined as any clinical or laboratory complication occurring after gene therapy infusion, with particular attention to events during the first month of follow-up. These included clinical manifestations, laboratory abnormalities, and the need for additional interventions, as documented in clinical records.

Continuous variables were described using measures of central tendency and dispersion, and categorical variables as absolute and relative frequencies. Due to the small sample size, no inferential analyses were performed, and results were presented descriptively.

The study protocol was approved by the institutional research ethics committee (CAAE: 58482122.9.2002.5558; opinion no. 5.622.863). Legal guardians provided written informed consent, and all procedures followed the principles of the Declaration of Helsinki.

## Results

### Sample characteristics

Fourteen families of children with type 1 and type 2 5q SMA were initially recruited, of whom seven maintained active clinical follow-up through June 2025. Five patients were female (71.4%). Six children had type 1 5q SMA and one had type 2 disease. A family history of SMA was identified in one case.

The mean gestational age at birth was 38 weeks, with a predominance of cesarean delivery (5/7; 71.4%). The median Apgar score at both the first and fifth minutes was 9. Reduced fetal movements were reported in three pregnancies (42.9%).

Symptom onset occurred before six months of age in children with type 1 SMA and between 12 and 15 months in the child with type 2 disease. The most frequent initial manifestations were hypotonia (5/7; 71.4%), muscle weakness (4/7; 57.1%), delayed motor development (3/7; 42.9%), and swallowing disorders (3/7; 42.9%).

### Genetic characteristics and pre-infusion clinical status

All participants presented a homozygous deletion of the *SMN1* gene. The number of *SMN2* copies ranged from two to three: six children had two copies and one had three copies. Individual clinical, genetic, and pre-infusion functional characteristics are detailed in Table 1.

**Table 1:** Pre-infusion clinical, genetic, and care characteristics of patients with 5q SMA.

Patient	5q SMA type	SMN2 copy number	Age at infusion	Pre-infusion motor status	Pre-infusion respiratory support	Pre-infusion nutritional support	Former Therapies (Doses)	Concomitant Therapies (Post-infusion)
P1	Type 1	2	1y2m	AC	NIV	GT	Nusinersen (6 doses)	None
P2	Type 1	2	1y2m	AC	Tracheostomy	GT	Nusinersen (8 doses)	Risdiplam
P3	Type 1	2	1y3m	SWS	NIV	GT	Nusinersen (3 doses)	None
P4	Type 2	3	1y3m	SWS	Room air	NGT	Nusinersen (3 doses)	None
P5	Type 1	2	1y4m	HCS	NIV	GT	Nusinersen (5 doses)	None
P6	Type 1	2	1y9m	Partial HCS	Room air	NGT	Nusinersen (7 doses)	None
P7	Type 1	2	2y	Partial HCS	Room air	NGT	None	Nusinersen

AC, absent head control; HCS, complete head control; Partial HCS, incomplete head control; SWS, sitting with support; Room air, breathing without ventilatory support; NIV, noninvasive ventilation; Tracheostomy, tracheostomy ventilation; GT, gastrostomy tube; NGT, nasogastric tube.

### Gene therapy administration

Onasemnogene abeparvovec-xioi infusion was administered in a hospital ward in five children (5/7; 71.4%). The mean age at treatment was 1 year and 5 months, ranging from 1 year and 2 months to 2 years. All patients received prophylactic corticosteroid therapy prior to infusion according to the institutional protocol. Post-infusion follow-up was conducted by a multidisciplinary team different from the one responsible for drug administration.

### Adverse events and safety

Six children (6/7; 85.7%) experienced at least one adverse event during the first month after gene therapy infusion. Most events were mild to moderate, with nausea and/or vomiting being the most frequent. There was one case of elevated liver enzymes that did not require corticosteroid dose adjustment and one case of thrombotic microangiopathy, which required hospitalization and dialysis, with favorable clinical recovery after supportive treatment. The detailed distribution of adverse events during the first post-infusion month is presented in Table 2.

**Table 2:** Adverse events observed within 30 days after onasemnogene abeparvovec-xioi infusion.

Adverse event	n (%)
Nausea/vomiting	3 (42.9)
Thrombocytopenia	2 (28.6)
Fever	1 (14.3)
Increased airway secretions	1 (14.3)
Elevated liver enzymes	1 (14.3)
Thrombotic microangiopathy	1 (14.3)

### Health care utilization

After the first month following infusion, five children (5/7; 71.4%) required evaluation in an urgent care unit, predominantly due to pneumonia (4/7; 57.1%) and less frequently due to swallowing disorders (1/7; 14.3%). Four patients (4/7; 57.1%) required hospitalization, with pneumonia as the main cause (3/7; 42.9%), followed by respiratory failure (1/7; 14.3%).

### Motor evolution

Compared with the pre-infusion evaluation, subsequent assessments showed a reduction in the frequency of fasciculations, present in 5/7 patients (71.4%) before infusion and in 1/7 (14.3%) after treatment. Polymyoclonus decreased from 2/7 (28.6%) to 1/7 (14.3%). Improvement in axial function was observed, with head control present in 6/7 patients (85.7%), although incomplete in 2/7 (28.6%).

**Figure 1:**
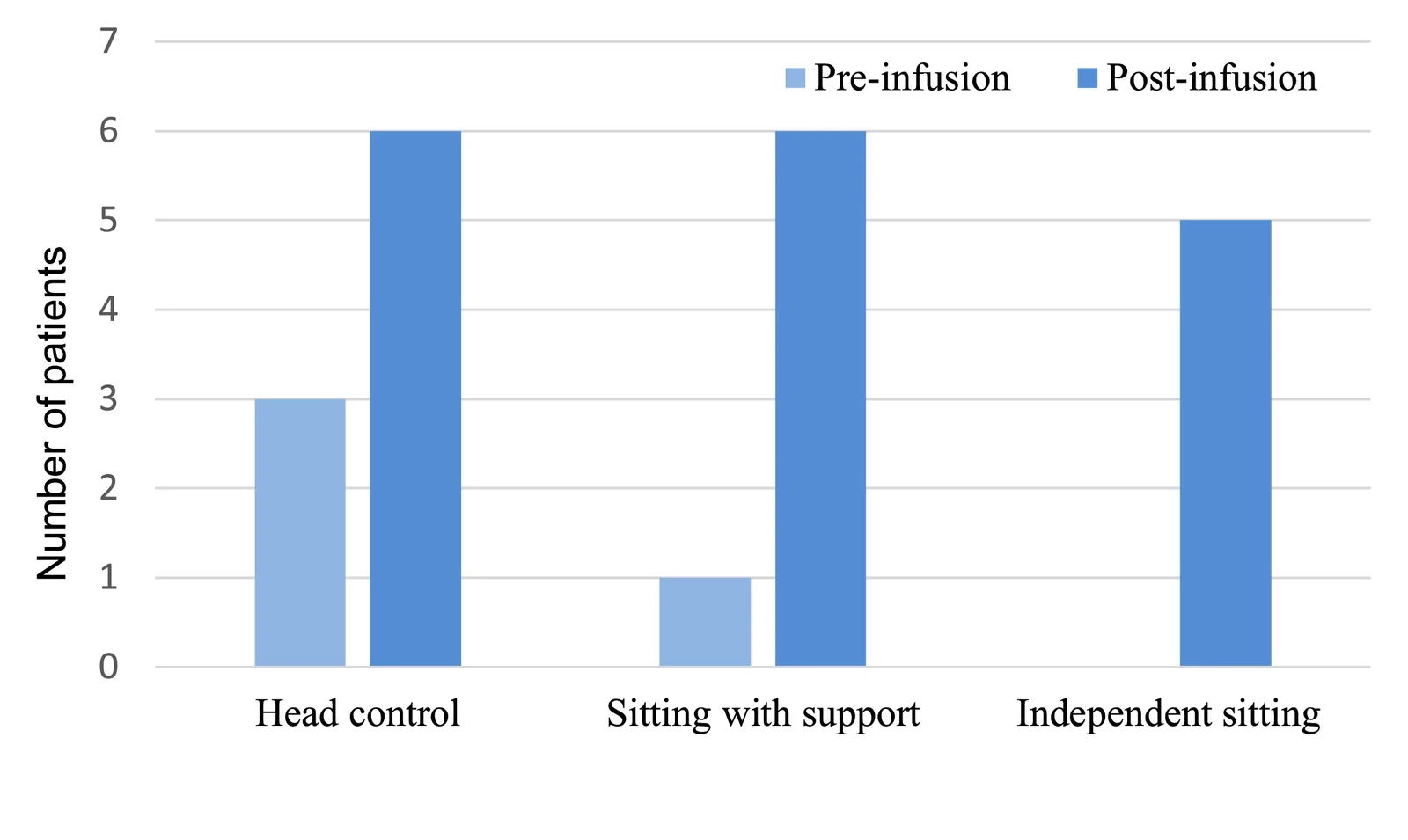
Motor milestone evolution in patients with type 1 5q spinal muscular atrophy before and after gene therapy.

Among patients with type 1 5q SMA (n = 6), all maintained head control and acquired the ability to sit with support, and 5/6 (83.3%) achieved independent sitting. The progression of motor milestones in this group is shown in Figure 1. The patient with type 2 5q SMA demonstrated broader functional improvement, achieving independent ambulation during clinical follow-up.

### Motor scale assessments

Motor function was evaluated using the CHOP-INTEND, HINE-2, and HFMSE scales according to age and functional capacity. The first assessment was performed after a post-infusion follow-up interval and clinical evaluation by the study team. Individual post-infusion results are presented in Table 3. Two patients were assessed using both scales at different time points after gene therapy due to functional progression and changes in motor profile over the course of follow-up.

**Table 3:** Post-infusion motor scale scores.

Patient	Age at infusion	Age at assessment	Scale	Score
P1	1y3m	5y2m	CHOP-INTEND	39
P2	1y9m	4y9m	HINE-2	8
		5y5m	HINE-2	8
P3	1y2m	3y4m	CHOP-INTEND	34
		3y4m	HINE-2	5
P4	1y3m	4y9m	HFMSE	51
P5	2y	3y4m	CHOP-INTEND	36
		3y4m	HINE-2	9
P6	1y4m	3y11m	CHOP-INTEND	37
		4y6m	CHOP-INTEND	36
		3y11m	HINE-2	11
		4y6m	HINE-2	9
P7	1y2m	4y3m	CHOP-INTEND	34
		4y3m	HINE-2	8

CHOP-INTEND: Children's Hospital of Philadelphia Infant Test of Neuromuscular Disorders.

HINE – 2: Hammersmith Infant Neurological Examination, Section 2.

HFMSE: Hammersmith Functional Motor Scale Expanded.

### Respiratory function and swallowing

After infusion, six children (6/7; 85.7%) required noninvasive ventilation. Use of manual resuscitation bag ventilation decreased from three to two children. The number of patients with tracheostomy increased from one to two following a respiratory complication.

Regarding swallowing, in the pre-infusion period four patients (57.1%) used gastrostomy feeding, and only one (14.3%) had partial oral feeding. After gene therapy, one patient (14.3%) achieved exclusive oral feeding and four (57.1%) had partial oral intake combined with gastrostomy. The number of gastrostomy-fed patients increased to six (85.7%), of whom two (28.6%) depended exclusively on enteral nutrition.

### Estimated therapy costs

All patients had received nusinersen prior to gene therapy (3–8 doses). During post-infusion follow-up, three initiated risdiplam and one restarted nusinersen. Estimated individual treatment costs were calculated and are presented as supplementary material due to therapeutic heterogeneity and the exploratory nature of this analysis.

## Discussion

In this real-world observational study, children with 5q spinal muscular atrophy (SMA) treated with onasemnogene abeparvovec-xioi after symptom onset showed preservation of motor function and limited acquisition of motor milestones. Specifically, patients with type 1 SMA achieved foundational milestones such as head control, whereas the single patient with type 2 SMA demonstrated broader functional improvement. These findings should be interpreted with caution due to the descriptive nature and small sample size of this cohort. However, they align with previous real-world data from larger registries, such as the RESTORE registry, which similarly note that while gene therapy modifies the clinical course leading to functional stabilization, complete neuromotor recovery is rarely achieved when treatment is initiated after significant symptom onset [10].

The observed motor response is biologically plausible. Gene therapy enables SMN protein expression in surviving motor neurons but does not restore neurons that have already degenerated. Consequently, patients treated after symptom onset tend to show clinical stabilization and modest functional gains, in contrast to the more robust outcomes reported in clinical trials and observational studies involving presymptomatically treated patients [8,11]. This mechanism explains the pattern observed in this cohort, in which patients with type 1 SMA achieved milestones such as head control and improved trunk control but did not progress to more complex motor abilities.

Despite motor improvement, the need for respiratory and nutritional support remained high throughout follow-up. The persistence of noninvasive ventilation and gastrostomy reflects established bulbar and respiratory impairment, which is often only minimally reversible when treatment begins after these dysfunctions are present. Previous studies indicate that even after disease-modifying therapies, symptomatic patients frequently remain dependent on respiratory and nutritional support, reinforcing the importance of ongoing multidisciplinary care [12].

The isolated interpretation of gene therapy effects in this cohort is strictly limited and cannot be generalized. A primary limitation is the very small sample size (n = 7), which precludes inferential statistical analysis and broader extrapolation. Additionally, notable patient heterogeneity (including both type 1 and type 2 phenotypes) combined with the prior or simultaneous use of other disease-modifying therapies, such as nusinersen and risdiplam, represent significant confounding factors inherent to real-world practice [13]. The lack of a concurrent control group further restricts our ability to isolate the definitive effect of the gene therapy itself. Nevertheless, this work contributes valuable, objective descriptive data regarding the outcomes and high supportive care needs of symptomatic patients in a real-world scenario, underscoring that therapeutic expectations must remain realistic when treatment begins after symptom onset.

In symptomatic children with 5q SMA, treatment with onasemnogene abeparvovec-xioi was mainly associated with clinical stabilization and limited motor milestone acquisition, without significant reversal of respiratory and nutritional impairment. These results underscore the importance of early treatment and continued multidisciplinary follow-up after infusion.

## Generative AI and AI-assisted technologies in the writing process

During the preparation of this manuscript, the authors used ChatGPT (OpenAI) to assist with English language translation and improvement of clarity and readability of the text. After using this tool, the authors reviewed and edited the content as needed and take full responsibility for the content of the published article.

## Affiliation for index Medicus/MEDLINE registration

University Hospital of Brasília, University of Brasília, Brasília, Brazil.

## Conflicts of interest

The authors declare no conflicts of interest.

## Data Availability

The data that support the findings of this study are available from the corresponding author.

## Appendix Supplementary materials

Image, application 1
