# Supplementary material for: Gene therapy benefits in a small cohort of symptomatic 5q spinal muscular atrophy patients and real world pediatric outcomes
Source: J Pediatr (Rio J). 2026 Sep 5;102(6):101597. doi: 10.1016/j.jped.2026.101597 (PMC13571994; doi:10.1016/j.jped.2026.101597)
Supplement: Supplementary file 1 [file mmc1.docx]

**JPED-D-26-00083_Supplementary Material**

**Supplementary Table** Individual estimated costs associated with disease-modifying therapies in patients with 5q spinal muscular atrophy (USD).

| Patient | Nusinersen doses | Nusinersen cost (USD) | Risdiplam months | Risdiplam cost (USD) | Gene therapy cost (USD) | Total cost (USD) |
| --- | --- | --- | --- | --- | --- | --- |
| P1 | 3 | 249,116.37 | NR | 0 | 1,400,000.00 | 1,649,116.37 |
| P2 | 4 | 249,116.37 | NR | 0 | 1,400,000.00 | 1,649,116.37 |
| P3 | 6 | 332,155.16 | NR | 0 | 1,400,000.00 | 1,732,155.16 |
| P4 | 3 | 249,116.37 | NR | 0 | 1,400,000.00 | 1,649,116.37 |
| P5 | 7 | 581,271.53 | NR | 0 | 1,400,000.00 | 1,981,271.53 |
| P6 | 8 | 664,310.32 | 26 | 1,351,678.22 | 1,400,000.00 | 3,415,988.54 |
| P7 | 5 | 664,310.32 | NR | 0 | 1,400,000.00 | 2,064,310.32 |

NR: not received.
